# The Art of Grid Fields: Geometry of Neuronal Time

**DOI:** 10.3389/fncir.2016.00012

**Published:** 2016-03-08

**Authors:** Andrey L. Shilnikov, Andrew Porter Maurer

**Affiliations:** ^1^Neuroscience Institute and Department of Mathematics and Statistics, Georgia State UniversityAtlanta, GA, USA; ^2^Institute for Information Technology, Mathematics and Mechanics, Lobachevsky State University of Nizhni NovgorodNizhni Novgorod, Russia; ^3^Department of Neuroscience, McKnight Brain Institute, College of Medicine, University of FloridaGainesville, FL, USA

**Keywords:** grid cells, theta, phase resetting, navigation, oscillators

## Abstract

The discovery of grid cells in the entorhinal cortex has both elucidated our understanding of spatial representations in the brain, and germinated a large number of theoretical models regarding the mechanisms of these cells’ striking spatial firing characteristics. These models cross multiple neurobiological levels that include intrinsic membrane resonance, dendritic integration, after hyperpolarization characteristics and attractor dynamics. Despite the breadth of the models, to our knowledge, parallels can be drawn between grid fields and other temporal dynamics observed in nature, much of which was described by Art Winfree and colleagues long before the initial description of grid fields. Using theoretical and mathematical investigations of oscillators, in a wide array of mediums far from the neurobiology of grid cells, Art Winfree has provided a substantial amount of research with significant and profound similarities. These theories provide specific inferences into the biological mechanisms and extraordinary resemblances across phenomenon. Therefore, this manuscript provides a novel interpretation on the phenomenon of grid fields, from the perspective of coupled oscillators, postulating that grid fields are the spatial representation of phase resetting curves in the brain. In contrast to prior models of gird cells, the current manuscript provides a sketch by which a small network of neurons, each with oscillatory components can operate to form grid cells, perhaps providing a unique hybrid between the competing attractor neural network and oscillatory interference models. The intention of this new interpretation of the data is to encourage novel testable hypotheses.

## Introduction

“The world being made of both space and time, it proved impossible to evade embroilment in spatial patterns of timing.”

*—(Winfree, 1987b)*.

In the 1990’s, at the University of Arizona, Arthur T. Winfree was investigating the intersection of math and biology. While his research drew from diverse fields including the circadian rhythm of fruit flies, the Kreb cycle of yeast, three dimensional knotted and twisted waves, chemical reactions, and the propagation of activity through cardiac muscle, the themes were the same—*oscillations* (for a comprehensive review, see Strogatz, 2003). Specifically, he was interested in the coupling of biological oscillators. Moreover, he considered how relatively minute inputs could alter the state of the system, potentially destroying the rhythmicity. His attitude of being a “nomad by choice” was fruitful, providing both immense amount of intellectual insights into oscillatory coupling as well as garnering him multiple awards including the MacArthur “genius” grant in 1984. Unfortunately, Dr. Winfree passed away from a brain tumor in 2002 (Johnson, 2002; Glass, 2003; Tyson and Glass, 2004).

Nonetheless, as serendipity would have it, Art Winfree’s research may also extend into the present, with significant and profound implications for the discovery of grid cells, which earned Drs. May-Britt Moser, Edvard Moser and John O’Keefe the 2014 Nobel Prize in Physiology or Medicine (Fyhn et al., 2004; Hafting et al., 2005; Moser and Moser, 2008; Burgess, 2014; Kandel, 2014; Fenton, 2015). This manuscript will delve into the confluence of these two parallel streams of research, although it should be noted that the potential existed for them to converge much sooner. In fact, while Dr. Winfree contemplated the geometry of oscillators using markers, cut-outs and graph paper often distorted to make a torus, an attractor network of neurons, arranged into a torus was being implemented in a laboratory across campus at that exact moment of time in order to provide in a model of hippocampal path integration (Samsonovich and McNaughton, 1997). Moreover, Dr. Winfree studied the Belousov-Zhabotinsky (BZ) reaction that gives way to periodic oscillations that can be seen with the naked eye, in order to understand the manner in which the waves spatially propagate as well as the excitability of the refractory zones (Winfree, 1972, 1973). Perhaps along a corresponding line of thought, Dr. Bruce McNaughton and colleagues considered the chlorite-iodide-malonic acid (CIMA) reaction as a potential mechanism that forms patterns akin to grid cells or striations in the visual cortex (McNaughton et al., 2006). The similarity between the two medium is that they are reaction-diffusion systems, a chemical oscillator, in which the temporal kinetics influence the spatial pattern (and vice versa).

Despite these near collisions (see Box 1), the field of hippocampal place cells and entorhinal grid cells did not reconvene with toroidal geometries until 9 years later (McNaughton et al., 2006). To our current knowledge, the phenomenon of grid cells has not been described in terms of *The Geometry of Biological Time* (Winfree, 2001) until the present manuscript. Nonetheless, integration of these two research streams and their implicit derivatives can now provide a novel theoretical perspective on the neurobiological underpinnings of grid cells and the role of oscillations in learning and memory.

Box 1 **The Bill Skaggs Connection.**

“In my copy of the 1980 printing of this book [The Geometry of Biological Time], the notion … that intrinsic inhomogeneity might be a red herring has a penciled notation beside it dated February 1988: ‘This is what Bill Skaggs and I seem to be finding while computing rotors in the Beeler and Reuter (1977) model of ventricular myocardium”’ (Winfree, 2001, 2nd edition)

While the physical proximity between Winfree and McNaughton on the University of Arizona campus may be coincidental, the potential intersection comes infinitesimally close as both laboratories independently attracted and trained Dr. William Skaggs. Moreover, in Bill Skaggs’ seminal publication providing a comprehensive investigation of theta phase precession (Skaggs et al., 1996), Art Winfree is acknowledged among a list of prominent of hippocampal researchers: “We thank John O’Keefe, Gyuri Buzsiki, Arthur Winfree, Misha Tsodyks, Mayank Mehta, Jim Knierim, and Alexei Samsonovich for helpful discussions”. Therefore, it is undeniable Bill Skaggs maintained a connection with Art while investigating hippocampus and plausibly brought Winfree’s theoretical toolbox with him. One may speculate that the toroidal idea traveled from one lab to the other with Bill as the conduit.

Bill Skaggs’, with a prepared mind’s eye to see a pattern in the noise, was adeptly tuned to identify the repetitious fields embedded in the now Nobel worthy discover of grid cells: “We particularly appreciate a breakfast meeting with Bill Skaggs at the Society for Neuroscience in 2004, where Bill drew our attention to the apparent hexagonal structure of the grids in the Fyhn article. Whether a periodic pattern was present could not be determined from the existing data; larger environments were needed” (Moser and Moser, 2008). Bill politely refused authorship on the manuscript, but inevitably utilized his training with Art Winfree to leave an indelible mark on the field.

## Properties of Grid Cells and Theoretical Models

“This geometrical context has been uniformly absent from discussions of phase resetting in circadian clocks, perhaps because no one has yet monitored the spatial pattern of timing after a resetting stimulus”

*— (Winfree, 1987a, p. 169–170).*

The discovery that hippocampal neurons fired action potentials that strongly correlated with the position of a rat in an environment (“place ells”; O’Keefe and Dostrovsky, 1971; O’Keefe and Conway, 1978; O’Keefe and Nadel, 1978), which earned Dr. John O’Keefe a half-share of the 2014 Nobel Prize, lead to a new branch of neuroscience research. As the activity of multiple place cells would cover an entire environment, changing their activity as a rat moved, O’Keefe went further to surmise that there may be another class of cells providing information “about changes in the direction of movement” (O’Keefe, 1976). Shortly after this, head direction cells were discovered by James Ranck, Jr. (Taube, 2009) with explicit characterization and description, cells that fire to a particular direction independent of location, completed by Taube et al. (1990a,b). The next few decades provided a wealth of information regarding the properties of hippocampal place cells and head-direction cells, along with a portion of studies sought to determine the spatial properties of the afferent input to the hippocampus from the entorhinal regions (Barnes et al., 1990; Mizumori et al., 1992; Quirk et al., 1992). As an interesting aside, a manuscript published by Quirk and colleagues, recording in the entorhinal cortex of freely behaving rodents, contains figures that can be reinterpreted in terms of the contemporary results—that is, the highly-regular, periodic fields of medial entorhinal neurons. Nonetheless, the explicit description was not uncovered until more than a decade later (Fyhn et al., 2004; Hafting et al., 2005). Multiple comprehensive reviews provide the history and implications of medial entorhinal grid cells (e.g., Hartley et al., 2013; Moser et al., 2014a,b) and will not be discussed here. It is however, necessary to discuss the primary models of grid cell formation traditionally divided into two classes; either a “Continuous Attractor Network” or “Oscillatory-Interference” class (Giocomo et al., 2011b). As both models have provided an immense amount of insight into potential mechanisms of grid cell formation, and importantly invoke conceptual parallels to the research of Art Winfree, it is necessary to discuss the unique features.

### Attractor Models: Why a Torus With a Twist?

A discussed in the dissertation of Bill Skaggs (1995), one way to construct an oscillatory network would be circularly connect non-oscillating neurons in a ring with unidirectional, excitatory (Dunin-Barkovskii, 1970) or inhibitory coupling (Morishita and Yajima, 1972). Such periodic activity would settle into a stable pattern continue to propagate around the ring with sequentially firing neurons; the group velocity is determined by temporal characteristics of individual neurons or synapses, or both, while period of the activity moves also depends on the size of the ring (the number of constituent neurons). The obvious advantages of such a network are that activity could be maintained indefinitely unless acted upon by an outside force, and that it provides periodic solution(s) to a circular phenomenon. Perhaps this is why, due to its spatial compactness, a similar one-dimensional ring attractor was also implemented to model the angular selectivity of head-direction cells. Head direction neurons, found in multiple regions of the brain (for review, see Dumont and Taube, 2015), are neurons that fire when a rat faces a particular direction in space. Following a rotation of 360°, the rat would have sampled every possible circular orientation available and theoretically activated every possible dedicated head-direction neuron. As the activity across head direction cells exhibits smooth continuous transitions from one neuron to the next, it has been suggested that anatomical organization of head direction cells completes a ring (McNaughton et al., 1991; Skaggs et al., 1995; Sharp et al., 1996b; Touretzky and Redish, 1996; Zhang, 1996; Knierim and Zhang, 2012). Theoretically, the firing rate across all head-direction cells would provide a population code (Maunsell and Van Essen, 1983; Georgopoulos et al., 1986), with the represented direction as the average of all activated neurons within the dedicated pool/array. Such a pool is associated with an “activity bump” on the head-direction ring that can be further shifted by the rat turning its head in either direction (Sharp et al., 1996b).

**Figure 1:**
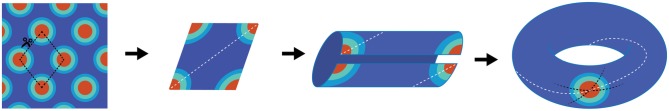
**Schematic depiction of why grid cells map to a torus.** The far left panel is cartoon depiction of a grid cell’s firing pattern in a two-dimensional environment. Making a cut through the center of four fields provides a diamond shape (second panel), which comprises the base unit. The dashed white line illustrates running the “long distance” in the diamond between the furthest two fields. By connecting the bottom edge to the top edge, two “half-fields” are generated (third panel) which becomes a single field when connecting the opened ends (far right panel). Note that there are three lines on the completed torus. The dashed black lines represent the original cuts along made in the first panel. The white line resembles the trajectory necessary to connect the two furthest points in the diamond. Note that it makes one revolution per rotation. That is, the trajectory travels through the interior of the tours as well as along the exterior.

The concept of a continuous bump attracter within the head-direction cell network was further extended for place cells too. Unlike head-direction cells, however, a population of place cells is presumed to evoke a continuous bump attractor defined on a two dimensional array/mesh subjected to periodic boundary conditions. For example, while a rat makes a complete turn from 0–360°, the head-direction neurons continuously fire to each orientation to maintain the activity bump propagating around the ring without interruption. With respect to place cells, a rat running on a linear track can also pass through multiple fields, theoretically without repetition. This begins a quandary in which the neural representation of extended bounded 2D space is finite, and hence must be limited by the total numbers of neurons and synaptic connections, whereas actual environmental space, for all intents and purposes, is infinite. If a rat were provided the opportunity to traverse a large environment, perpetually traversing unique terrain, eventually the rat’s physical location in the real environment would escape the boundaries of the neural, 2D bump attractor. Alternatively, in order to prevent real-world motion from resulting in “falling off the cognitive map” (O’Keefe and Nadel, 1978), a periodic boundary condition (aka “ring”) could be used to solve the linear track problem. Extending this to a 2D environment, the rectangular neural sheet would involve connecting the east and west boundaries together as well as stitching the north and south boundaries, effectively making a torus. The utilization of a torus to solve periodic boundary conditions has a long history in the field of mathematics, topology and physics and it is therefore difficult to find the initial novel use. In the field of dynamical systems, a torus is the phase space for a coupled system with two independent natural frequencies, such as in the case of synchronization of a pair of Christiaan Huygens’ clocks, hanging on a common bar. Given the ubiquity of the toroidal compactification of space, and the historical perspective outlined above, it may have been an intuitive extension of 1D attractors on the circle (math symbol S^1^) to continuous attractor networks in 2D neural fields on a torus (math symbol T^2^ = S^1^ ⊗ S^1^ a Cartesian product of two circles in topology). The topological consecutive of using torus in spatial navigation, however, implies that the firing patterns of neurons in this network should be either a square or a rectangle (McNaughton et al., 1996, 2006; Samsonovich and McNaughton, 1997). Rats running in a fixed direction would eventually return to the same position on the neural network, although occupy a physically different location in the real-world. Firing patterns of grid cells, of course, are not squares or rectangles, but rhomboidal. Therefore, in order to account for the tessellating diamond patterns (Hafting et al., 2005) a single twist to construct the torus needs to be added (Guanella et al., 2007; Figure 1).

All models are not without faults (or errors by exclusion) that can be modified in later iterations. Nonetheless, it is necessary to discuss the potential issues with a continuous attractor concept in order to consider its plausibility. While there is significant amount of support for the continuous attractor neural network model, many issues arise in terms of its biological plausibility. For example, this model necessitates a great deal of developmental complication, as it requires a pool of neurons that literally form a synaptic torus in the brain. Specifically, there would be thousands of neurons dedicated to the structure and support of a single torus, each with a specific size and spacing of grid fields. While this issue can tenably be settled via developmental mechanisms of Hebbian plasticity (Fuhs and Touretzky, 2006), the continuous attractor neural network model does not quite explain why grid cell activity is abolished following the attenuation of the theta rhythm (Brandon et al., 2011; Koenig et al., 2011), a prominent 4–12 Hz oscillation in the hippocampus and entorhinal cortex (Jung and Kornmüller, 1938; Green and Arduini, 1954; Vanderwolf, 1969).

Finally, this model suggests that there may be a “local vulnerability” in which, if a local region of grid cells with the same size, spacing, orientation and phase are obliterated, then it would compromise the entire network. Lesioning a significant number of cells would effectively punch a hole in the torus, possibly distorting the map. Therefore, it begs the question that, if biology works towards parsimonious solutions, would this be the developmental mechanism that supports grid fields?

### Oscillatory Interference Models

**Figure 2:**
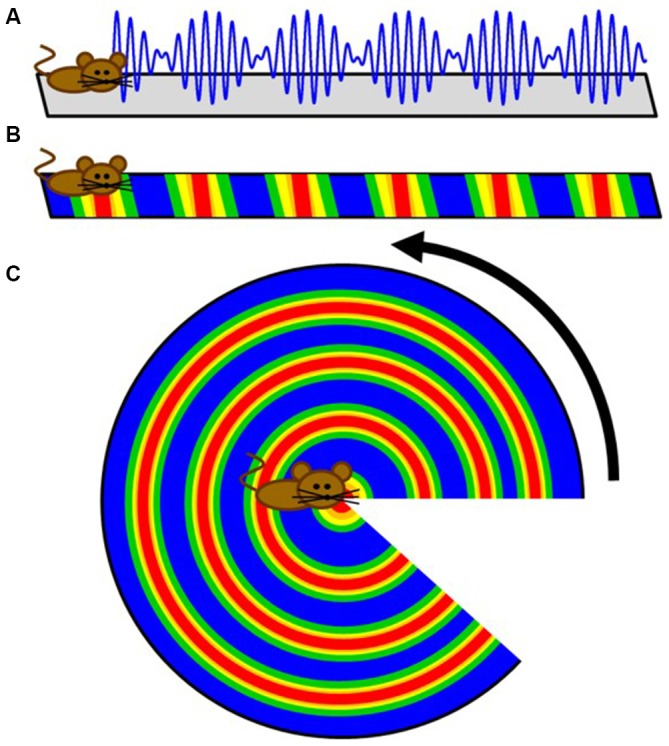
**Cartoon extension of a simple intrinsic oscillator model to two dimensions. (A)** Schematic depicting how the interference pattern between two oscillators would map onto a linear track. **(B)** The maximum amplitude locations of the interference pattern would translate to repeating fields on the linear track. **(C)** While these results are sufficient for linear traversals, this simple model does not directly translate to two dimensions as a rotation demonstrates. Therefore, more advanced controls were implemented to create the tessellating pattern observed in grid fields.

The first prediction of competing oscillations in the hippocampal formation may have been provided by John O’Keefe in 1985. Using the premise that the theta rhythm emerges from two inputs: (1) the afferent entorhinal projections onto the CA1 and dentate dendrites; and (2) the interneuron interaction with principal cell somas (Buzsáki et al., 1983), O’Keefe rationalized that there would be an interaction of “two theta systems on each cell… Only when the two theta waves had the correct phase relationship would a particular set of afferents have preferential access to a particular cell” (O’Keefe, 1985). The extension of this idea can be seen as a potential model description of theta phase precession, which demonstrated that a linear sum between two oscillations of different frequencies results in a harmonic envelope (O’Keefe and Recce, 1993; Figure 2A). While accumulating evidence suggested an oscillatory interference model was untenable for the hippocampus (for review, see Maurer and McNaughton, 2007), perhaps the most outstanding remainder of the model was that place field firing would wax and wane (oscillate in time or in space, or in both) indefinitely. In this circumstance, cells would have repeating fields that cease to turn off. With respect to hippocampal place cells, however, the number of fields per cell follows a logarithmic distribution, with only a few cells expressing more than three (Shen et al., 1997; Maurer et al., 2006; Mizuseki and Buzsáki, 2013; Buzsáki and Mizuseki, 2014). The discovery of grid fields, in which neuron activity repeated at regularly distributed/spaced intervals, however, resurrected the oscillatory models (O’Keefe and Burgess, 2005; Burgess et al., 2007; Giocomo et al., 2007). Moreover, the existence of subthreshold membrane oscillations in neurons in the medial entorhinal cortex (Alonso and Llinás, 1989; Dickson et al., 2000a,b; Fransén et al., 2004) provided a tenable oscillation that could “interact” with the local theta rhythm. The premise of the idea is that an interaction between distinct oscillators generates an interference pattern of tessellating grid fields. The interference of two oscillators, however—in the absence of directional control—are not sufficient to produce grid fields because the spatial patterns of activity produced from the interaction of just two oscillators in 2-dimensions would be concentric circles (Figure 2). Therefore, more advanced mechanisms were implemented in order to account for the periodicity seen in grid cells. These theories utilized the linear summation of multiple dendritic oscillators (O’Keefe and Burgess, 2005; Burgess et al., 2007; Hasselmo et al., 2007). In order to achieve a geometric pattern of firing, the dendritic compartments are set to oscillate with a preferred spatial periodicity in which each compartment would have a “band” of activity across the environment. The three bands, in order to linearly summate to form the geometry observed in grid fields, must be arranged with an exact 60° offset to each other (Burgess et al., 2007; Giocomo et al., 2007; Hasselmo et al., 2007; Zilli and Hasselmo, 2010). Otherwise, the fundamental grid shape would become distorted.

As elegantly demonstrated by Remme et al. (2010) and discussed by Fiete (2010), biological oscillators can often demonstrate more complex dynamics resulting from nonlinear interactions of coupled compartments/components other than due to their linear summation. Remme and colleagues revealed that the interaction between the dendrite and the cell body must be unrealistically weak in order for each oscillating compartment to maintain independence from the somal activity. Otherwise, as demonstrated in biologically plausible models of stellate neurons, each dendrite becomes strongly phase-coupled with each other and the soma (Remme et al., 2010). Therefore, while there is a significant amount of evidence that supports the role of intrinsic oscillations in the formation of grid field, the results on their own cannot fully explain the dynamic properties of grid cells.

## Art Winfree and the Geometry of Biological Time

### Phase Resetting, the Existence of Another Torus and Oscillatory Convergence

The intersection between grid cells and the research of Art Winfree potentially begins with the biological relevance of interacting oscillators, which dates back to the research of Norbert Wiener (Wiener, 1965; Strogatz, 1994). Truly fascinated by biological oscillators, Art Winfree sought to determine how multiple components act synergistically on each other for the sake of global synchrony without focusing on a single system (Winfree, 1967, 2001). While his visits to distinct research fields of biology were often short, intermittent or both, Winfree’s work tended to leave a significant theoretical impact—as stated by one of his mentees, Steven Strogatz: “Art Winfree has changed the way we think about several entire subfields of science… His brilliant intuitions have repeatedly opened new fields of mathematical inquiry” (Johnson, 2002).

**Figure 3:**
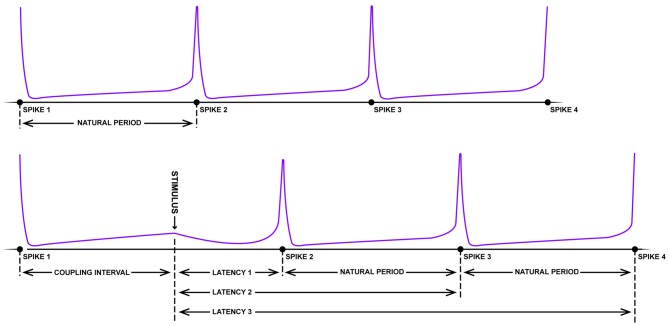
**Schematic of resetting for a periodic firing neuron.** The top panel depicts the rhythmic firing of an unperturbed neuron that fires four spikes with a near-fixed natural period. In the bottom panel, somewhere between the first and second spike, a strong pulse stimulus arrives which delays the onset of the next spike (latency). Following this disruption, however, the natural period resumes albeit shifted in time (figure adapted from Winfree, 1983).

With respect to the existence of grid fields, significant parallels begin appearing between the neurobiology of spatial navigation and phase response curves. Imagine the pendulum of a grandfather clock swinging rhythmically back and forth. If, with the lightest of touches, a feather is pressed against the swinging pendulum, little to no change in the phase of the pendulum will occur (independent of the phase that the collision occurs; type I resetting). On the other hand, a precocious toddler has the potential to dramatically reset the phase of the clock with a well-timed swipe. Large stimuli have the potential to reset the phase of the clock pendulum, with limited sensitivity to the initial phase of the oscillation at the time of impact (type 0 resetting). Using some well-crafted intuition, Art Winfree studied the pacemaker firing properties of neurons from a lobster, Aplysia, crayfish and cricket in 1977. As these neurons fire with a fixed, rather long duration between rapid spikes, it is possible to treat the spikes as an oscillation, assigning specific phases to the action potentials. Knowing the frequency of the firing and the time of the most recent spike, one can predict when the next spike will fire (Figure 3). Using this information, one can determine how the timing of small perturbations in a form of either inhibitory or excitatory short pulses, relative to the phase of the spiking neuron can delay or speed up (facilitate) the generation of the next spike. By plotting the unperturbed phase of the neuron spiking at the time of stimulation (“old phase”) against the perturbed or reset spike phase (“new phase”) for experimental data, the researcher can empirically calculate a phase resetting curves for the system (Winfree, 1977; Canavier et al., 2009), as well as assess the core features of dynamical equations with a stable limit cycle describing the spiking neuron.

**Figure 4:**
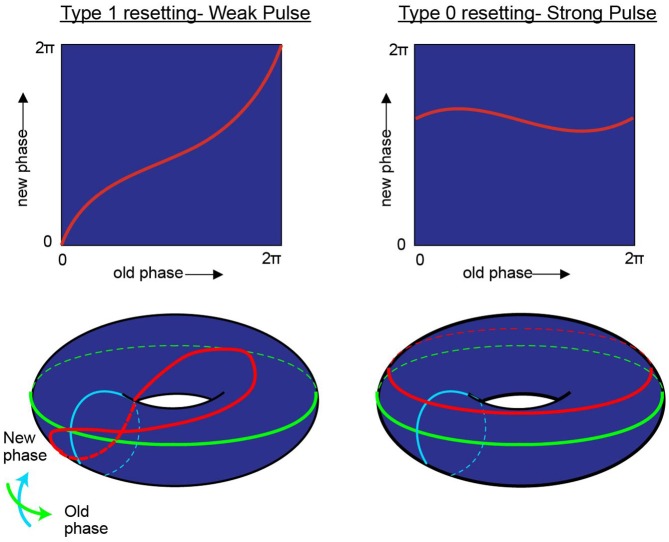
**While research on shifting the timing of spikes in pacemaker neurons is influential in its own right, it is likely to also be relevant to the oscillatory kinetics of the membrane potential for neurons in the medial entorhinal cortex.** In his investigation of pacemaker resetting, Art Winfree chose to plot the resetting curves onto a torus rather than a flat sheet. In this manner, the equatorial line, or green line on the doughnut plane would represent the old phase of the oscillation while the turquois line would represent the new phase. In the type 1 resetting situation, left column, a weak stimulus has little to no effect (new phase equals old phase), and the initial state and outcome are effectively the same. When plotted onto a torus (red line), this trajectory effectively runs from the exterior, transitioning to the top of the torus, through the interior, underneath and back to the start; the line effectively traces the topology one would expect from a twisted torus (Guanella et al., 2007). Alternatively, if the stimulus was sufficient to consistently reset the oscillator (right column) independent of the time it arrived, then the resetting curve would fail to venture through the center of the torus (figure modified from Tyson and Glass, 2004).

*“Viewed topologically, the new phase and the old phase are periodic coordinates, more properly represented along circles than along Cartesian coordinate axes, so the new phase-old phase plane is really the unrolled surface of a torus” (Winfree, 1977; Figure 4)*.

**Figure 5:**
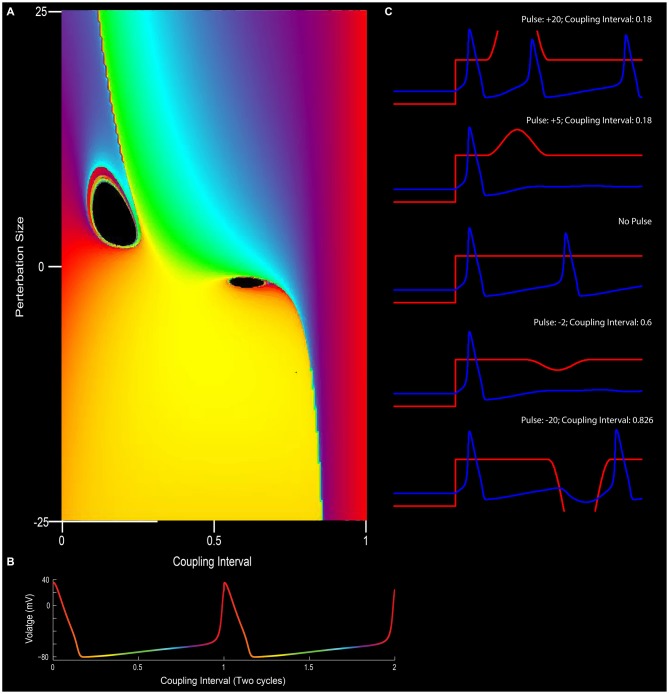
**The effect of phase resetting on the squid giant axon. (A)** For a pacemaker cell, the duration between spikes can be plotted in terms of “cycles” or coupling interval in which the first spike is 0 and the second spike is 1 (*x*-axis). An outside stimulus can arrive at any time between 0 and 1. In the circumstance in which there is no input, the cells “new phase” is equal to the old phase (0 point on *y*-axis). In this depiction, phase is color coded such that early points are purple/blue, mid-cycle (right in between two action potentials) is yellow, and the instance before an action potential is red. **(B)** Depiction of pacemaker firing, with color-coded phases between two action potentials (red). In **(A)**, modulating the input into a neuron in terms of excitation or inhibition (*y*-axis), can alter the phase-resetting of a neuron. **(C)** For example, very strong excitatory stimulation will inevitably drive the cell to fire (top panel; also the red section at the top left of **(A)**) and strong inhibition will often reset the neuron to earlier phases (bottom of **C** and yellow at the bottom of panel **A**). Small excitatory or inhibitory inputs, however, when well-timed, can put the neuron into a “singularity” (black holes) that blocks the rhythmicity/stops the clock. Note the cessation of firing for the +5 or −2 pulse conditions in **(C)** (See Best, 1979 and Winfree, 1983).

In the borderlines between type I (weak stimulus; the plot between the old and new phase still travels through the hole of the torus) and type 0 resetting (strong stimulus; new phase virtually remain constant and independent of when the stimuli are applied), there is a specific magnitude of perturbation that, when delivered at the appropriate time with respect to the position of the pendulum in swing that will completely halt all motion (phase singularity, also known today as a sudden death of oscillations; Winfree, 1970, 1987a; Glass and Winfree, 1984). By considering the Hodgkin-Huxley equation for the squid giant axon as a cycling system, Eric Best (graduate student of Art Winfree at Purdue University), sought to determine how the new vs. old phase relationship transitioned from type 1 to type 0. Specifically, in the transition from type 1 to type 0 phase resetting curves, Winfree and Best predicted the existence of a specific stimulus that, when delivered at a certain phase of a repetitively spiking squid giant axon would not result in consistent latencies. Specifically, there would be a specific stimulus that would induce a singularity, potentially abolishing rhythmic firing. Through simulation, the authors found that modest and appropriately timed input, whether it is inhibitory or excitatory, would prevent the pacemaker-like firing of the neuron from returning (Best, 1979; Winfree, 1983). By thoroughly investigating these results *in vitro* Guttman et al. (1980) confirmed the prediction that specific stimuli can cause a cessation in repetitive firing, supporting the hypotheses of Best (1979; Figure 5). Importantly, this research trajectory provided a comprehensive understanding on how neurons, either operating as pacemakers or oscillators, could reset their phase as a function of their input. In this manner, one can imagine the firing properties of a rhythmic neuron, perhaps a grid cell, to be embedded in such a synaptic matrix. The periodic firing of the neuron could be modified by its inputs, with the new and old phases together forming and living on a topological torus.

If there was a common theme to the research of Art Winfree, it would be the universal interest in the coupling between biological oscillators. In one facet of research, Winfree studied the process of glycolysis in yeast cells to determine how the phase of two oscillators may interact. Particularly, when breaking down glucose into pyruvate, Adenosine triphosphate (ATP) and Nicotinamide adenine dinucleotide (NADH) are produced, the latter of which, NADH, is fluorescent under Ultraviolet (UV) light. The production of NADH is synchronized across a population of yeast cells, with the fluorescence activity waxing and waning with a period of 30 s. Once provided with one oscillator, the temptation to add it to another must be overwhelming; simply take two populations of yeast cells at different phases of NADH production and add them together. Often, addition of the two populations would eventually synchronize although, for certain parameters, there were odd disparities in the data (Ghosh et al., 1971). Fortunately, using simple rules to conceptualize the problem, Art Winfree was able to discover an incongruity providing an important insight into the irreproducible results of Ghosh and his colleagues.

**Figure 6:**
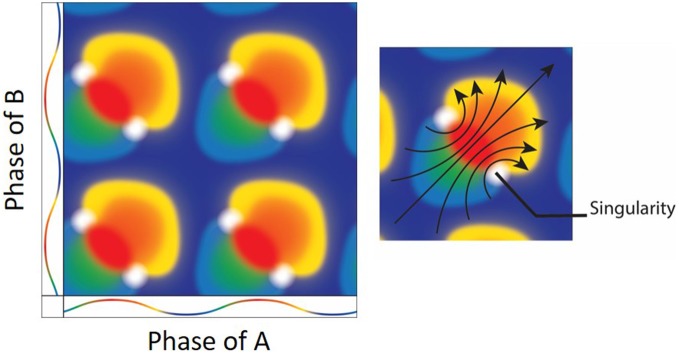
**(Left) The result of mixing two oscillators.** Both the *x* and *y* axes depict the color coded phases of two oscillators with the peak as red and trough as blue. Mixing the two oscillators when they are at the same exact phases results in a mixture of the same (e.g., red + red = red). There are, however, specific phase combinations that result in phase singularities (white). (**Right**) Interestingly, there is a directionality/rotation to the singularities. As the oscillation moves from blue at the trough, to red at the peak and back to blue, the geometrical depiction of the mixing shows a clockwise (bottom-right) and counter-clockwise (upper-left) rotors (arrows; Figure adapted from Winfree, 1987a).

Consider a situation in which two oscillators are mixed or converged. Each oscillator has a respective quantity, which can be treated as a “volume” if the oscillators were a liquid or “amplitude” if the oscillators were neurobiological. If we know the quantity of the two oscillators along with their respective phases, then one may predict or expect that slightly changing/perturbing the phase of either oscillator and recombining it with the original will only mildly and linearly alter the outcome. This simple assumption, however, may not be always true. Similar to spiking recorded from the squid giant axon, there are specific combinations of input in terms of magnitude and spike timing that can produce unexpected or nonlinear outcomes or even cause a collapse in rhythmicity (see Box 2). The collapse in the example of yeasts results in two singularities (Figure 6). That is, when there are two populations of yeast cells added to each other, there will be a distinct combination that will yield a distorted phase outcome. As described by Art Winfree: “What happens at these singularities? The system won’t stay at an ambiguous phase after the critical stimulus, but neither is it allowed to have any predictable phase, so it becomes unpredictable” (Winfree, 1987a). Surrounding this singularity, the phase of the resulting system could land in all possible “in-between” states (for a mathematical description, please see Winfree, 1974). This is akin to the amphidromic point of ocean tides. On a water-laden planet with no continents, high tide would occur on the side adjacent to the moon as well as on the side diametrically opposed. Traveling around the equator, we would experience all tide phases twice—two high tides, two low tides and everything in between. Moving towards the poles, however, we would eventually discover the amphidromic point, where like time zones, all of the tide levels simultaneously converge and vanish/annihilate. Surrounding theses amphidromic points, the tide height “rotates” in either a clockwise or counter-clockwise direction (“rotary tides”). This rotation is exactly what manifests in the missing of the yeast oscillators. Given that these rotors have specific turning directions, it would plausibly introduce a specific distortion if mapped onto dimensions outside of time (space perhaps; see below). It should be noted that the depiction of two mixing oscillations could also be described in terms of two oscillators, with the resulting colors as the “skin” of the torus, analogous to Figure 1. Moreover, this short review into the research of biological oscillators provides the frame work necessary to stitch together parallels between Grid Cells and The Geometry of Biological Time (Winfree, 2001).

Box 2 **The Winfree Model of Phase Compromise.**

When considering the interaction of two or more coupled oscillators, the simplest Winfree model can be described by the equation: θi’ = ω − ɛP(θj) · Q(θi), mod 1 where ɛ describes the coupling strength. By construction, the phase space of such a system is a 2D torus. When uncoupled, the phase of each oscillator grows with speed equal ɛ (the frequency of the oscillator). Here the function P represents the phase resetting curve (PRC) that describes how the phase oscillator responds to small perturbations that can either speed up or slow down its phase. These perturbations come from the other oscillator(s). With a certain choice of *P*, a pair of such oscillators will produce robust anti-phase oscillations, like a half-center oscillator, so that the average phase lag between the oscillators is 1/2. Typically, this would correspond to reciprocal inhibitory coupling between neurons. In the excitatory case, the neuron would oscillate in-phase. The grid cells can be also viewed as oscillators that produce a specific firing field with specific and robust phase lags between the constituent neurons (perhaps under more complex equations as described in Winfree, 1974). Adopting a more complex model, the phase of grid cells would plausibly be controlled by theta modulated interneurons and theta-cycle-skipping head direction cells (see Figure 9)

## The Spatial Geometry of Biological Time

Integrating the grid cell phenomenon with what Art Winfree and colleagues noted regarding the convergence of oscillators in other biological systems results in two significant departures from the previously proposed models. The first is that the torus with a single twist only exists as a conceptualization that occurs when one plots one oscillation against another—that is, while it is probable that there is a continuous attractor mechanism, there is no “homunculus torus” in which each neuron in the medial entorhinal cortex occupies a specific node. Rather, the toroidal geometry is a function of biological time. Biological time can be defined on the most general level as the rhythmic activity that organizes the processes that support life (Winfree, 1987a). In terms of neuroscience, biological time can be described as the complex dynamics, which tend to result in a plethora of oscillatory patterns in support of basic functions to complex cognition (Buzsáki and Draguhn, 2004). With respect to the interaction among grid cells, biological time considers all potential phases of one oscillator against all phases of another oscillator will inevitably construct a 2D torus (with the unity phase moving from the exterior, through the center and returning through the other side and back to the start adding the twist; bottom left panel of Figure 4). Time and space become interchangeable in the nervous system (at least of primitive animals). Moreover, the problem of collapsing an infinite system (space) into the finite representation contained within the nervous system is solved by the interaction between two or more oscillators. A rat can convert an infinite amount of territory, charting its spatial progress via the temporal offset between two cells (effectively coupling time and space; Buzsáki, 2013).

**Figure 7:**
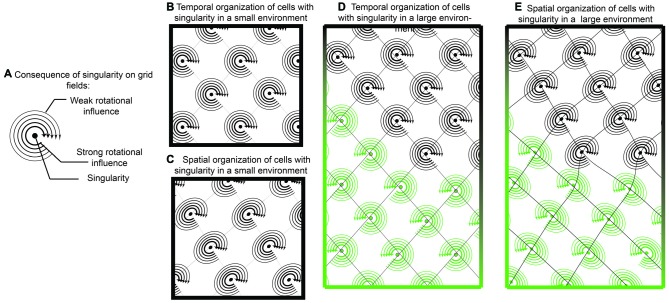
**Singularity and shearing of grid cells. (A)** If grid cells were the result of oscillatory interaction with only one singularity, then they could tenably have a “rotational direction” in which it is easier to update or “phase reset” the neuron. Locations closer to the singularity could be updated easier than locations farther away. **(B)** While temporally, the competition between two oscillators would resemble traditional grid cells, when translated onto space, there would be a mild but consistent distortion **(C)**. In this sense, a local cue in the environment provides a strong influence over one of the oscillators. While this distortion may be governed by a single cue in small environments, it is possible that two distinct cues have differential effects in larger environments **(D,E)**. In this example, one environmental cue results in a clockwise rotation, with shearing in one direction while another cue results in counter clockwise rotation.

The second major departure is that the integration of oscillators may not be a linear summation, but nonlinear aggregation able to yield beautifully complex patterns. While models of dendritic oscillations are most likely untenable (Remme et al., 2010), stellate neurons of layer II of the entorhinal cortex are endowed with I_h_ channels providing them with the ability to oscillate near theta frequency. Therefore, stellate neurons are potential candidates for facilitating intrinsic pacemaking oscillations (Alonso and Llinás, 1989; Dickson et al., 2000b; Fransén et al., 2004). Specifically, as they sit in an oscillating medium, neurons may interact with each other via synaptic influence as well as ephatic coupling (Radman et al., 2007; Anastassiou et al., 2011). Therefore, the extracellular medium as well as inhibitory and excitatory synapses can interact on single stellate neurons to modulate the neurons’ oscillatory phase. Given this framework, it should appear tenable that two oscillators are sufficient to support grid field firing patterns (for example, see Hasselmo, 2008; Figure 6). Specifically, the geometrical representation of two oscillators mixing results in the repeating “fields,” which follows from the toroidal geometric representation. Implementing this for grid cells, one needs to consider that the frequencies of the oscillations do not need to be equal and that oscillations do not have to be sinusoidal. Moreover, the amplitudes of each oscillator do not need to be equal, potentially altering the singularity as well as the overall geometry of the system (which was discussed in Winfree, 1987a). Applying this to grid fields, by altering the frequencies or amplitudes between the two oscillators could effectively interact to “phase reset” the grid cell in a trajectory specific manner. Another important point to note is that the specific mixing of two oscillators can possibly result in a phase singularity in time. This temporal singularity in oscillatory interactions would also be present in the spatial periodicity of the neurons—a position in space in which the phase of the neuron is indeterminate akin to an amphidromic point in the ocean. Just outside of the geometrical singularity, the spatial alignment of the oscillation—moving from 0 to 360°—follows a specific rotation direction being either clockwise or counter clockwise (Figure 6). It should be noted that output of Figure 7, the nonlinear interaction of sinusoidal oscillations, do not form equilateral triangles/repeating rhomboids observed in grid cells (rather, this simulation produces squares). One potential avenue to consider when attempting appropriately achieve tessellating triangles is to consider that the oscillations may not be sinusoidal. That is, the underlying oscillatory dynamics may be more akin to “saw-tooth” waves (nonlinear oscillations; Leung, 1982; Buzsáki et al., 1985; Leung and Yu, 1998). The consequence being that the nonlinear interactions of nonlinear waves would make skewed phase resetting curves with fields repeating in a rhomboid manner. Moreover, Figure 7 is the temporal relationship (not spatial). Therefore, the mapping from time-to-space may not necessarily be 1-to-1, but distorted, plausibly generating a pattern more akin to grid fields.

Continuing in this framework, tenably, phase resetting dynamics may occur easier if the rat were moving in a trajectory that coincides with the rotation direction. If the rat was moving in a trajectory against the rotor (rotor defined as the spatial rotation around the singularity), it would theoretically be more difficult to update the grid cell phase of firing. This is one tenable mechanism responsible for the recently observed shearing phenomenon of grid fields, an elliptical distortion in the grid pattern (Krupic et al., 2015; Stensola et al., 2015). As the grid cell shearing effect is experience-dependent, it is worth considering that the combinatorial influence of two oscillators in grid cells is: (1) synaptic; and (2) capable of undergoing spike-time dependent plasticity. In this sense, neurons that synapse onto grid cells, potentially border cells or head-direction cells strongly anchored to walls, may have a dominant influence over grid cells and how the intrinsic oscillation phase is updated with locomotion (Knight et al., 2011, 2013; Clark et al., 2012). With a rotor and unequal contribution, potentially due to differences in amplitude, oscillatory convergence may result in grid cell distortion (Figure 7).

In this vein, unequal contribution of environmental cues may be the reason for the fragmented representation with repetitive and redundant activity of grid cells in a hair-pin maze (Derdikman et al., 2009). As the strength and timing of inputs are the factors that determine how the phase of a neuron is updated (Best, 1979; Winfree, 1987a), one possibility is that small movements with minimal velocity have limited influence on the phase resetting (type 0 resetting). A conceptually similar related idea has been presented by Michael Hasselmo to describe the fragmented fields in a hairpin maze (Hasselmo et al., 2007; Hasselmo, 2008). In open fields, however, similar hairpin trajectories failed to result in fragmentation (Derdikman et al., 2009).

*“Stability of the antiphase equilibrium is independently rediscovered almost annually in new contexts, presumably starting long before my own encounter with it, and continuing still in 1999.” (Winfree, 2001; p. 123)*.

While the nonlinear interaction of oscillators in this context may be able to explain the properties of grid cells, it does not offer much in the way regarding the neurobiological connections and synapses that give rise to the phase resetting of grid cells. Specifically, what is the architecture of the network and where do the oscillators reside? A unique intersection between half-center oscillators, antiphase equilibrium, and “cycle skipping” may provide some further parallels into the mechanism of grid cells activity. Again, this requires a bit of explanation prior to developing the integration.

The half-center oscillator concept introduced by Brown (1911) involves two non-oscillatory (or non-endogenously bursting) neurons, coupled to each other via inhibitory synapses (as cited Sharp et al., 1996b). As discussed earlier, the spike timing of one neuron will alter the timing of the other neuron and vice versa. The two will push on each other, thereby resulting in a few potential outcomes, which include synchronous as well as antiphase firing/bursting (Coombes and Lord, 1997; Winfree, 2001). Moreover, fast reciprocal inhibition can synchronize bursting neurons (Wang and Buzsáki, 1996; Jalil et al., 2010; Assisi and Bazhenov, 2012). When extended to larger groups of oscillators, some units that are coupled to each other will fire together while there will be overall antiphase synchronization across two different subgroups or clusters (Kawamura, 2014); potentially analogous to the 180° phase preference offset between interneurons in the hippocampus (Klausberger et al., 2003), which can even coexist with antiphase synchronization among three subgroups that would correspond to the so-called traveling waves with phase 120° shifts or phase lags (Wojcik et al., 2011). To understand the importance of half-center oscillators, it is necessary to take a look at their function in terms of contemporary modeling efforts.

**Figure 8:**
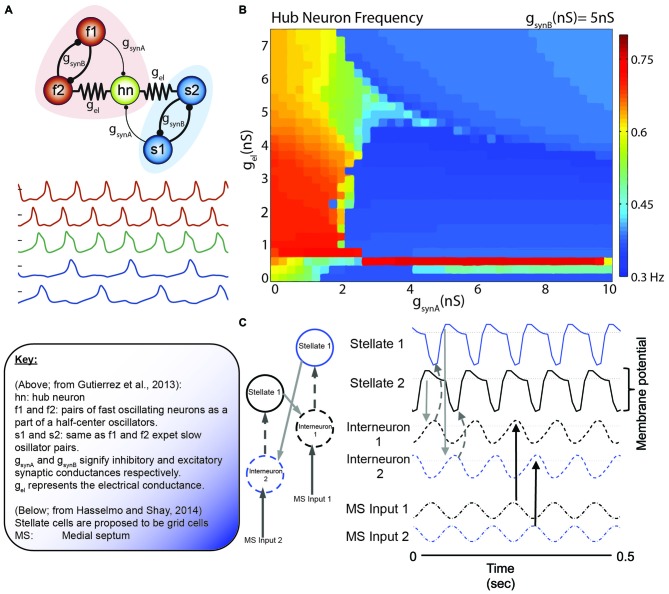
**The collective behavior of a system of coupled oscillators. (A)** Model circuit of interconnected neurons in the gastric (blue) and pyloric (red) of the Jonah Crab. Below are the model voltage traces, by color for each neuron, in the network. Note that the f1/f2 pair and s1/s2 pair are half center oscillators. In this specific simulation, g_synA_ is weak relative to the other connections (thin lines). **(B)** Hub neuron frequency as a function of synaptic conductance. While the hub neuron traditionally has an intrinsic frequency of 0.57 Hz, altering the afferent activity can alter the oscillatory dynamics. Also, note the similarity in the continuity (and discontinuity) in the frequency shifts relative to the phase resetting curves of Best (1979; Figure 5). **(C)** Incredibly, similar models are coming to light in terms of grid cells. Here, Grid cells (stellate neurons) are interconnected with interneurons that receive antiphase afferent drive from the medial septum. Hasselmo and Shay independently converged on the use of half-center oscillators, although not directly connected, in order to modulate stellate cell oscillatory frequency. Figures adapted from Gutierrez et al. (2013) and Hasselmo and Shay (2014).

In a meticulous modeling study Gutierrez et al. (2013) illustrated the dynamics of a small, five neuron network in the Jonah crab (*C. borealis*) that exhibit significant parallels to the grid cell network of the rodent**.** The problem as poised by Gutierrez et al. echoes a similar sentiment to the quote of Art Winfree (1987a) at the start and is foundational to the organization of grid cells: “how are individual neurons or groups of neurons switched between, or recruited into, different oscillatory networks as a function of the strength of the electrical and chemical synapses in the network?” (Gutierrez et al., 2013). The authors discussed the mechanisms by which different cellular mechanisms can account for alterations in neuronal function, facilitating recruitment and information transfer through oscillatory coupling. More importantly, oscillatory frequency can also be modulated in these models. Specifically, oscillation frequency is directly coupled to electrical and chemical synapses embedded into a network that can achieve the same pattern of activity through multiple initial states (degeneracy). Using the somatogastric ganglion neural network, Gutierrez et al. (2013) investigated the respective contribution of electrical and chemical junctions between neurons of this small network in modulating the activity of the IC pyloric neuron (known as the “hub neuron” in the models). The elegant take home of this comprehensive effort is that subtle interactions of electrical and chemical synapses are capable of altering the oscillation frequency of the IC pyloric neuron (Figure 8). Importantly, similar activity could result across a range of parameters with degenerate circuit mechanisms capable of achieving the similar output. In order to advance the current state of the field, it is important to note that the gastric-pyloric network model takes advantage of fast and slow oscillatory pairs, “half-center oscillators,” that are out of phase with each other (Sharp et al., 1996b). The slow oscillatory pair in this model is half the frequency of the fast oscillator.

This model draws strong similarities to the neurons in the medial entorhinal cortex. Simply, the IC pyloric neuron may be similar to grid cells and the fast-half center oscillators as pairs of ~8 Hz modulated, 180° out of phase interneurons. The slower, ~4 Hz half-cycle oscillator would theoretically be carried by the theta-cycle skipping head direction cells in the entorhinal cortex. Brandon and colleagues suggest that there are two populations of these neurons, exchanging firing bouts on every other theta cycle. Moreover, these neurons exhibit large differences in their head-direction tunings, often with differences larger than 40° (Brandon et al., 2013). Utilizing theta-skipping head direction cells in order to modulate grid cell firing is not a novel idea, but was recently implemented by Hasselmo and Shay (2014; Figure 7). This model is unique in that it directly emphasizes the roles of resonance and rebound excitation in the interaction of neurons.

*“Quite often it matters little what your guess is; but it always matters a lot how you test your guess” (George Polya, 2014)*.

**Figure 9:**
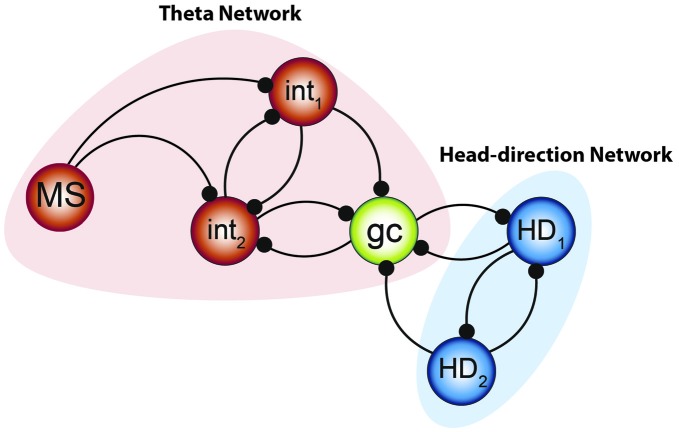
**Integrated model to be tested.** The combination of the two systems of oscillators by Gutierrez et al. (2013) along with the small network constructed by Hasselmo and Shay (2014) suggests that the “smallest unit” of grid cell activity may potentially resemble the network aligned above. Medial septal neurons pace the interneuronal network, forming the fast- half-center oscillator—while the theta cycle skipping head direction neurons form the other half of the network. Their convergence onto the grid cell can effectively pace the intrinsic oscillation and, when combined with the input from other grid cells, effectively path-integrate.

The above quote was a favorite of Winfree appearing in a few of his books. While a good deal of conjecture will follow, it is our hope that it will stimulate new theories in how to test phase resetting models of grid cells in attractor networks. This, at best, is a skeleton schematic presenting one potential organization of synapses, in a small network, which supports the nonlinear oscillator interactions presented above. Nonetheless, by integrating Art Winfree’s ideas with more contemporary research from crabs and rats, it is plausible to conceive of a network capable of complex dynamic patterns. Specifically, we hypothesize that grid cells are organized into networks of coupled oscillator with complex nonlinear interactions, capable of producing multiple activity patterns (such as the periodicity observed in foraging vs. the regularity seen in hair-pin mazes) and perhaps shearing (see below). Without mathematically formalizing the approach, it may be possible to provide a “back-of-envelope” sketch by integrating what is already known regarding the observed properties of grids cells with the ideas of Eve Marder and colleagues (Sharp et al., 1996b; Gutierrez et al., 2013) with those of Hasselmo et al. (2007; Figure 9). In this extension, the IC pyloric neuron becomes the grid cells, the fast half-center oscillators are interneurons and the slow half-center oscillators are theta cycle-skipping head direction neurons. This small model swaps out components, but conserves a good deal of the connectivity. One advantage of utilizing half-center oscillators is that the bursting/oscillatory patterns seen in the entirety of the network is not the result of endogenous cellular properties (Alaçam and Shilnikov, 2015), perhaps providing insight regarding why grid cells were sustained following I_h_ channel knockout (Giocomo et al., 2011a).

The strong scaffolding of the interneurons may also provide the connectivity to push and pull on the stellate oscillators, updating their timing in an appropriate manner (Buzsáki and Chrobak, 1995; Melzer et al., 2012). As local parvalbumin positive interneurons receive input from multiple grid cells, thereby providing recurrent inhibition, interneurons may be instrumental in the local coordination of the network (Buetfering et al., 2014; Hasselmo and Shay, 2014). While these synapses could be traditionally thought of as inhibitory, they could also serve to synchronize neurons in clusters (Cobb et al., 1995; Belykh and Shilnikov, 2008), shift their oscillatory phases with respect to each other (Jalil et al., 2012), or perhaps push the neural dynamics into a singularity (Best, 1979). In line with this idea, recent theoretical work has demonstrated that interconnected interneurons can functionally interact to control the mean period of activity, modulating the wavelength of periodicity as a function of excitation (Tristan et al., 2014). Moreover the resonance properties of stellate neurons depends on the I_h_ current (Dickson et al., 2000b; Fransén et al., 2004; Tristan et al., 2014), which can give rise to rebound spiking (Alonso and Llinás, 1989). Therefore, the resonance and activity of the neuron can be paced as well as influence the other neurons in the network. In earlier studies of half-center oscillators Sharp et al. (1996a) discovered that modulating the conductance of I_h_ could alter the neuronal period, supporting the finding that HCN1 channel knock-out mice have larger, farther spaced grid fields (Giocomo et al., 2011a).

This skeleton model integrates two components, theta and head direction neurons, with grid cells as the site of convergence. There are multiple pathways by which this input converges, either arising from the mammillary bodies (Vann and Aggleton, 2004), septal and anterior thalamic input, or a combination. The head-direction circuitry (arising from the medial mammillary bodies and anterior thalamus) would serve to provide the slower half-center oscillator to pace the grid cell oscillation. The faster oscillatory input, the theta rhythm, would be governed by medial septal input (Petsche et al., 1962; Stumpf et al., 1962). Moreover lesion or inactivations of the septum severely depress the hippocampal theta rhythms, resulting in spatial memory deficits and loss of CA3 place cell activity (Winson, 1978; Mizumori et al., 1989). Importantly, the skeleton model can explain why grid cells and conjunctive cells lose their spatial periodicity following septal inactivation (Brandon et al., 2011; Koenig et al., 2011), but also account for the mechanism by which conjunctive cells revert to simple head direction cells under these conditions. Moreover, as the theta-skipping head direction cells maintain their directionality but lose their theta modulation following septal inactivation (Brandon et al., 2013), the skeleton model suggests that grid cells operate as the primary mechanism to convey the rhythmicity to the head direction network. It does not imply that theta LFP is needed for the existence of grid cells, but that half-center oscillatory activity is necessary (Sharp et al., 1996a; Gutierrez et al., 2013). In light of this, grid cell activity should be dependent on head-direction activity. This prediction has recently been observed and following lesions or inactivation of the anterior thalamic nuclei, tenably relaying head-direction input to the medial entorhinal cortex (Winter et al., 2015), grid field patterns (i.e., the phase resetting) were severely disrupted. Moreover, this phenomenological model predicts that lesions of the head-direction cell network should affect memory to the same degree as lesions of the theta network. Another interesting aspect of utilizing this sort of network architecture is that it allows the occurrence of more complex, nonlinear dynamics, beyond the traditional single attractor. Specifically, a multifunctional network, i.e., possessing several coexisting attractors unlike a continuous attractor model, may allow the system to be more maneuverable, supporting rapid but deliberate changes in the dynamics (Schwabedal et al., 2014), and perhaps responsible for the changes in grid cell geometry observed in the hairpin maze (Derdikman et al., 2009). Moreover, the recent discover of periodic band cells, which can change between grids and nongrid cells across environments (Krupic et al., 2012; c.f., Navratilova et al., 2016), can be plausibly achieved by changing the contributions of each oscillator. That is, the periodic islands of phase resetting (Figure 6) can switch to bands depending on the quantity/strength of the interacting oscillators (Winfree, 1987a; p. 136–138). Finally, as demonstrated by Gutierrez et al. (2013), changes in one aspect of the activity may or may not have a profound influence-permitting metastable or/and multistable dynamics—and that similar changes in the intrinsic oscillation of a grid cell can be achieved via various/different degenerate mechanisms. As there are multiple oscillators that fire off cycle, including interneurons that exchange volleys of 8 Hz, there is a possibility that harmonics (16 Hz) may appear in the local-field potential at higher activity levels.

This theory, however, is conjecture that remains to be tested. Moreover, the circuit itself may be incomplete and plausibly requires other grid cells, with similar theta and head-direction inputs, to maintain appropriate updating characteristics. Moving forward, the intention is that this model is useful in shaping future experiments. Of course, as discussed by Ploya, the model matters very little when considered in the scope of how it is tested. We also hope that the perspective of Art Winfree is considered as the neurobiological investigations on grid fields move forward as he most certainly left other impactful discoveries yet to be related. We must apologize as this manuscript focused on the work of Art Winfree although there were significant contemporaries and influences who thought along the same lines—including Norbert Wiener, Collin Pittendrigh, Yoshiki Kuramoto, Theodosios Pavlidis, Rutger Weaver, Jurgen Aschoff, and Victor Bruce. Moreover, his legacy is continued forward by his students.

“From the growth of lichens on rocks, to the delicate patterns of chemical waves, to fatal cardiac arrhythmias, Art Winfree delighted in finding unexpected connections between geometry and dynamics. As researchers develop new ways to visualize the patterns of excitation in chemical systems and heart tissue, Winfree’s predictions about the initiation, geometry and stability of spiral waves and scroll waves are being tested and are yielding new insights”

*—Leon Glass*.

## Author Contributions

APM conceptualized the ideas and wrote the manuscript. ALS provided key early insights on the history and the phenomenon, provided critical comments through revisions and provided mathematical descriptions.

## Funding

The McKnight Brain Research Foundation (UF), University of Florida Seed Opportunity Fund and R03AG049411.

## Conflict of Interest Statement

The authors declare that the research was conducted in the absence of any commercial or financial relationships that could be construed as a potential conflict of interest.
